# Effectiveness of cognitive remediation in subjects with major depressive disorder: A multicenter randomized controlled study in a real-world setting

**DOI:** 10.1192/j.eurpsy.2025.10073

**Published:** 2025-08-04

**Authors:** Stefano Barlati, Gabriele Nibbio, Antonello Bellomo, Bernardo Carpiniello, Cristina Colombo, Serafino De Giorgi, Giacomo Deste, Giuseppe Maina, Giovanni Martinotti, Alfonso Tortorella, Gabriele Di Salvo, Mario Luciano, Federica Pinna, Antonio Ventriglio, Andrea Fiorillo, Antonio Vita, the CogRemMDD Study Group

**Affiliations:** 1 Department of Mental Health and Addiction Services, ASST Spedali Civili of Brescia, Brescia, Italy; 2 Department of Clinical and Experimental Sciences, University of Brescia, Brescia, Italy; 3 Department of Clinical and Experimental Medicine, University of Foggia, Foggia, Italy; 4 Section of Psychiatry, Department of Medical Sciences and Public Health, University of Cagliari, Cagliari, Italy; 5 Department of Clinical Neurosciences, University Vita-Salute San Raffaele, Milan, Italy; 6 Department of Mental Health, ASL Lecce, Lecce, Italy; 7 Department of Mental Health and Addiction Services, ASST Valcamonica, Esine, Italy; 8 Department of Neurosciences Rita Levi Montalcini, University of Torino, Turin, Italy; 9 Department of Neurosciences, Imaging and Clinical Sciences, Università degli Studi G. D’Annunzio, Chieti, Italy; 10 Department of Psychiatry, University of Perugia, Perugia, Italy; 11 Department of Psychiatry, University of Campania “L.Vanvitelli”, Naples, Italy

**Keywords:** cognition, cognitive rehabilitation, cognitive remediation, cognitive training, depression, RCT

## Abstract

**Background:**

Cognitive impairment represents a central component of major depressive disorder (MDD), affecting a large proportion of people living with MDD and showing a consistent negative impact on social, interpersonal, and occupational functioning and subjective quality of life. Cognitive remediation (CR) is a training-based psychosocial intervention targeting cognitive performance and psychosocial functioning that has shown consistent evidence of effectiveness in individuals with schizophrenia and that could provide significant benefits also in people with MDD: this study aimed to assess the effects of a computerized CR intervention in adults living with MDD.

**Methods:**

Participants recruited in this single blind multicentric randomized controlled trial were allocated to receive a computerized CR intervention delivered by an active and trained therapist or to an active control condition (computer games – CG). Outcomes were measured with validated instruments by blind assessors and included cognitive performance, depressive symptoms, and psychosocial functioning. Outcomes were assessed using mixed models for repeated measures, considering baseline and end-of-treatment scores.

**Results:**

Hundred and one participants (CR=52 and CG=49) were included and 81 (CR=45 and CG=36) completed the study. CR produced superior results in clinician-rated depressive symptoms (*p*=0.023, *d*=042), global clinical severity (*p*=0.025, *d*=0.39), subjective depressive symptoms (*p*=0.005, *d*=0.45), working memory performance (*p*=0.004, *d*=0.34), executive functions/cognitive flexibility (*p*=0.020, *d*=0.43), and subjective cognitive impairment (*p*=0.006, *d*=0.48).

**Conclusions:**

CR represents an effective intervention in MDD, improving clinical outcomes and cognitive performance in a clinician-rated and in a subjective manner, which should be more consistently implemented in clinical practice and included in MDD treatment recommendations.

## Introduction

### Background

Major depressive disorder (MDD) represents one of the most common mental health conditions on a global level, showing a lifetime prevalence of over 20% in high-income countries and interesting more than 300 million people worldwide [1–3]. It also represents an essential source of disability, producing a significant socio-economic burden that extends well beyond direct healthcare costs [4].

Cognitive impairment represents a central component of MDD [5–7]. Despite the high level of heterogeneity of clinical presentations of the disorder, cognitive impairment affects a large proportion of people with MDD, often reaching moderate levels of severity and emerging from the first depressive episode [8–10]. This impairment frequently persists after the remission of the affective episode and appears to increase in severity over time with relapses and with age [11, 12], involves both “hot” and “cold” cognitive functions [13–15] and appears to be composed of state, trait, and scar elements [5, 16, 17]. Processing speed appears to be the most heavily interested domain, showing also a state-dependent effect during affective episodes, but attention, memory, and executive functions are also involved [6, 18, 19].

Cognitive deficits in MDD represent one of the core determinants of impairment in social, interpersonal, and occupational functioning and also have a significant negative impactive on subjective quality of life [20–24]. As such, they are becoming a treatment target of increasing importance and interest [2].

Cognitive remediation (CR) is a training-based psychosocial intervention targeting cognitive performance to provide significant and durable improvement in psychosocial functioning [25]. Based on the structured repetition of cognitive exercises and the development of novel strategies with the help of an active and trained therapist [26], CR interventions have been shown to provide substantial positive effects on cognitive performance and on psychosocial functioning in people living with schizophrenia [27–31] which are durable over time [32]. As such, it currently represents the psychosocial intervention with the highest level of recommendation in the European Psychiatric Association guidance for the treatment of cognitive impairment in schizophrenia [33].

CR interventions appear as a promising treatment also in MDD: recently, four meta-analyses have specifically investigated this topic [34–37]. The first meta-analysis included 8 studies and 268 adults with MDD and reported positive treatment effects on most cognitive domains [37]. A meta-analysis included 19 studies for a total of 944 participants with MDD or clinically relevant and depressive symptoms and included both a superiority and a noninferiority analysis: CR emerged as effective in treating depressive symptoms and noninferior to standard interventions for depression [36]. Another meta-analysis included studies on individuals with affective disorders for a total of 22 randomized controlled trials, 4 of which of individuals with bipolar disorder, and 993 participants: it reported small to moderate positive treatment effects on cognitive performance and depressive symptoms [34]. Finally, another meta-analysis included only MDD studies and included 21 trials and 978 participants: significant positive effects were observed on all outcomes, medium-sized on cognitive performance, and small-sized in depressive symptoms and daily functioning [35].

While the results of these works converged in many aspects and showed that CR can represent an effective intervention in MDD, they also highlighted the current limitations in the available evidence: the certainty in the evidence assessed by Legamaat et al. with the GRADE procedure was very low for depressive symptoms and daily functioning, and low for cognitive performance [35].

Besides the high level of heterogeneity in participants’ characteristics, interventions, and measures included in the trials, most available studies included only small samples and were conducted in a single research center. Moreover, subjective experiences of participants and patient-reported outcomes measures, which are becoming increasingly important in a regulatory and in a treatment personalization perspective [38], were seldom considered in available trials.

In this perspective, a large multicentric randomized controlled trial, adopting standardized and validated tools, including patient-reported outcomes, could offer valuable insights into cognitive, depressive, and psychosocial outcomes in MDD from both scientific and clinical perspectives.

### Aims

The aims of the present single blind, multicentric randomized controlled trial were to assess the effects of a computerized CR intervention delivered by an active and trained therapist compared to an active control condition (computer games) in adults living with MDD undergoing a Major Depressive Episode (MDE) on cognitive performance, depressive symptoms, and psychosocial functioning. The main research hypothesis was that CR could provide significant improvements in all these outcomes and that the positive effects would be observed in both external, investigator-rated measures and in subjective, participant-reported measures.

## Methods

### Study design and participants

The present trial included nine different centers for the recruitment of participants: I) Department of Mental Health and Addiction Services, ASST Spedali Civili di Brescia and Department of Clinical and Experimental Sciences, University of Brescia, which also acted as the coordinating center; II) Department of Psychiatry, University of Campania Luigi Vanvitelli, Naples; III) Department of Clinical and Experimental Medicine, University of Foggia; IV) Department of Medical Sciences and Public Health, Psychiatry Section, University of Cagliari; V) Department of Clinical Neurosciences, IRCCS San Raffaele, Milan; VI) Department of Mental Health, ASL Lecce; VII) AOU San Luigi Gonzaga, Orbassano and Department of Neurosciences, University of Turin; VIII) Ospedale Santa Maria della Misericordia and Division of Psychiatry, Clinical Psychology and Rehabilitation, Department of Medicine, University of Perugia; IX) Department of Neurosciences, Imaging and Clinical Sciences, “Gabriele D’Annunzio” University of Chieti-Pescara.

Centralized training for the delivery of the CR and the control intervention and for the administration and rating of all outcome measures for researchers of all participating centers took place before the start of the study. Regularly scheduled online meetings were held during the course of the study between the coordinating center and all participating centers to assess and resolve potential issues.

Participants were consecutively recruited in each center with the following inclusion criteria: I) diagnosis of MDD according to DSM-5 criteria [39] confirmed with the Structured Clinical Interview for DSM-5- Clinical Version (SCID-5-CV) [40]; II) age between 18 and 60 years; III) clinical diagnosis of current MDE of mild or moderate severity according to DSM-5 criteria; IV) education of at least 8 years; V) consent to participate in the study, attested by a signed written informed consent form.

Exclusion criteria were: I) history of neurological disorders or severe traumatic head injury; II) diagnosis of intellectual disability; III) history of manic or hypomanic episodes, schizophrenia spectrum disorders or other psychotic disorders; IV) MDE with psychotic features and/or treatment resistance; V) history of alcohol or substance abuse in the previous 6 months; VI) participation in any type of CR intervention in the previous 6 months; VII) concurring pharmacological treatment with mood stabilizers, and first or second generation antipsychotics (benzodiazepines or Z-drugs low dose treatments were accepted); VIII) pregnancy.

Both outpatients and inpatients in psychiatric rehabilitation settings were considered for inclusion, as long as they met all inclusion criteria and no exclusion criteria. Participants were consecutively enrolled in each center from the study start (April 2021) to the recruitment of the targeted number of participants for each center, based on the results of a power analysis conducted prior to the start of the study.

All individuals who were considered for enrollment received a detailed explanation of the study aims and procedures. The subject agreed to participate in the study, provided explicit consent, and signed a dedicated written informed consent form.

The study was approved by the Local Ethical Committee (registration code NP 3173, approved April 29, 2020) of the coordinating center and of other participating centers and conducted in accordance with the Code of Ethics of the World Medical Association and the Declaration of Helsinki.

All necessary precautions were adopted to maintain patients’ anonymity and data confidentiality.

Participants were considered as drop-outs if they suspended antidepressant pharmacological treatment for more than 5 days and expressed intention of discontinuing it or if they did not participate in the active or control interventions for 2 consecutive sessions and expressed intention of discontinuing it.

Central randomization using a random number table was carried out in order to allocate participants to the CR intervention group or to the control condition. The randomization was performed by a researcher who was not involved in the delivery of the interventions, in the assessment of participants and in the data analysis.

### Interventions

#### Cognitive remediation intervention

The CR intervention (CR group) was delivered with a computerized approach, using the Cogpack program Version 6.0 (Marker Software®, Mannheim, Germany). The program is composed of several cognitive exercises, including domain-specific exercises as well as non-domain-specific exercises focusing on different domains at once. It also includes exercises that focus on language and calculation skills. The exercises can be adapted for individual participants, with the software automatically setting the difficulty level on the basis of the participant’s performance during the session.

The exercises were delivered with the assistance and feedback of dedicated therapists who were specifically trained to deliver the intervention for the present study. Therapists were trained in centralized seminars that included lectures and practical exercises and were supported throughout the duration of the study with regularly scheduled online meetings.

Therapists had an active role in the delivery of the cognitive exercises, providing instruction and feedback to participants, closely monitoring their progress, and fostering the development of novel cognitive strategies.

The cognitive exercises were also integrated into a structured rehabilitation program that included discussion sessions dedicated to the development of cognitive strategies, with the aim of improving the transfer of cognitive gains into real-world functioning.

The CR intervention was administered two times a week, in 45-minute sessions, for 12 weeks and a total of 24 sessions. Missed sessions that were not related to study dropout were individually rescheduled.

#### Control intervention – computer games

The control intervention, included to assess the role of placebo and nonspecific effects, consisted of the use of computer games (CG group). Available games were “Minesweeper,” “Solitary” and “Pinball,” according to the participant’s choice. Games were played in a setting that included the presence of the therapist, but in this instance, the therapist did not play any active role, and no discussion session on cognitive strategies was provided.

The control intervention was also scheduled two times a week, in 45-min sessions, for a duration of 12 weeks and a total of 24 sessions.

At the conclusion of the study and of the dedicated assessments, participants allocated to the control group were invited to receive the CR intervention.

### Measures

All participants were assessed at baseline (T0) and at the conclusion of the intervention (T1) with validated measures of cognitive performance, clinical symptoms, and psychosocial functioning.

Assessors were specifically trained to conduct the assessment and were kept blind as regard to the group allocation of participants for the whole duration of the study. Participants were explained and explicitly reminded not to disclose their group allocation to assessors.

Performance-based cognitive measures included the California Verbal Learning Test (CVLT) [41], measuring the domain of verbal learning and memory; the Digit Symbol Substitution Test (DSST) [42] measuring mostly processing speed; Trail Making Test A (TMT-A) and B (TMT-B), and B-A (TMT-B-A) [43] measuring processing speed, working memory, and executive functions/cognitive flexibility, respectively. Participant-rated measures included the Perceived Deficits Questionnaire – Depression 5 items (PDQ-D-5) to assess subjective cognitive dysfunction in people with depression [44].

Clinical severity measures included the Montgomery–Åsberg Depression Rating Scale (MADRS) [45] and the Clinical Global Impression – Severity scale (CGI-S) [46] for clinician-rated measures and the Beck Depression Inventory, Second Edition (BDI-II) [47] for participant-rated measures. Real-world psychosocial functioning was measured with the Personal and Social Performance scale (PSP) [48].

The inter-rater reliability was evaluated for all measures and for all assessors of each participating center after the centralized training using Interclass Correlation Coefficient (ICC): good to excellent agreement between raters was observed for all measures (ICC ranging from 0.62 to 0.91).

### Statistical analyses

All individuals recruited in the study and randomized to one of the treatment arms (CR group or CG group) who completed the initial assessment were included in the analyses according to their allocation, regardless of their completion of the study, to implement an Intention-To-Treat (ITT) approach.

Baseline socio-demographic (age, gender, years of education) and clinical characteristics were compared between groups using *t*-tests for continuous variables and *χ* ^2^ tests (or Fisher’s Exact test where appropriate) for categorical variables to identify potential differences: variables that emerged as significantly different were introduced as covariates in the outcome analyses to avoid potential confounding factors.

Drop-out rates were compared between the intervention groups to identify potential sources of bias. Baseline values for all outcome measures were also compared between study completers and dropouts.

Outcomes were assessed using mixed models for repeated measures (MMRM), considering baseline (T0) and end-of-treatment (T1) scores and group allocation (CR or CG) to implement an ITT approach. Statistically significant Time x Group interactions were considered as indicating a significant treatment effect. Simple effects for each treatment arm were also investigated.

*p*-Values <0.05 were considered significant. Analyses were performed using Jamovi version 2.2.5.

Between-group effect sizes were calculated as Cohen’s *d*, considering both baseline and end-of-treatment values and baseline standard deviations, and taking into account group allocation and size [49].

Statistical analyses were carried out maintaining blinding as regard to participants’ group allocation to avoid potential sources of bias.

## Results

**Figure 1.**
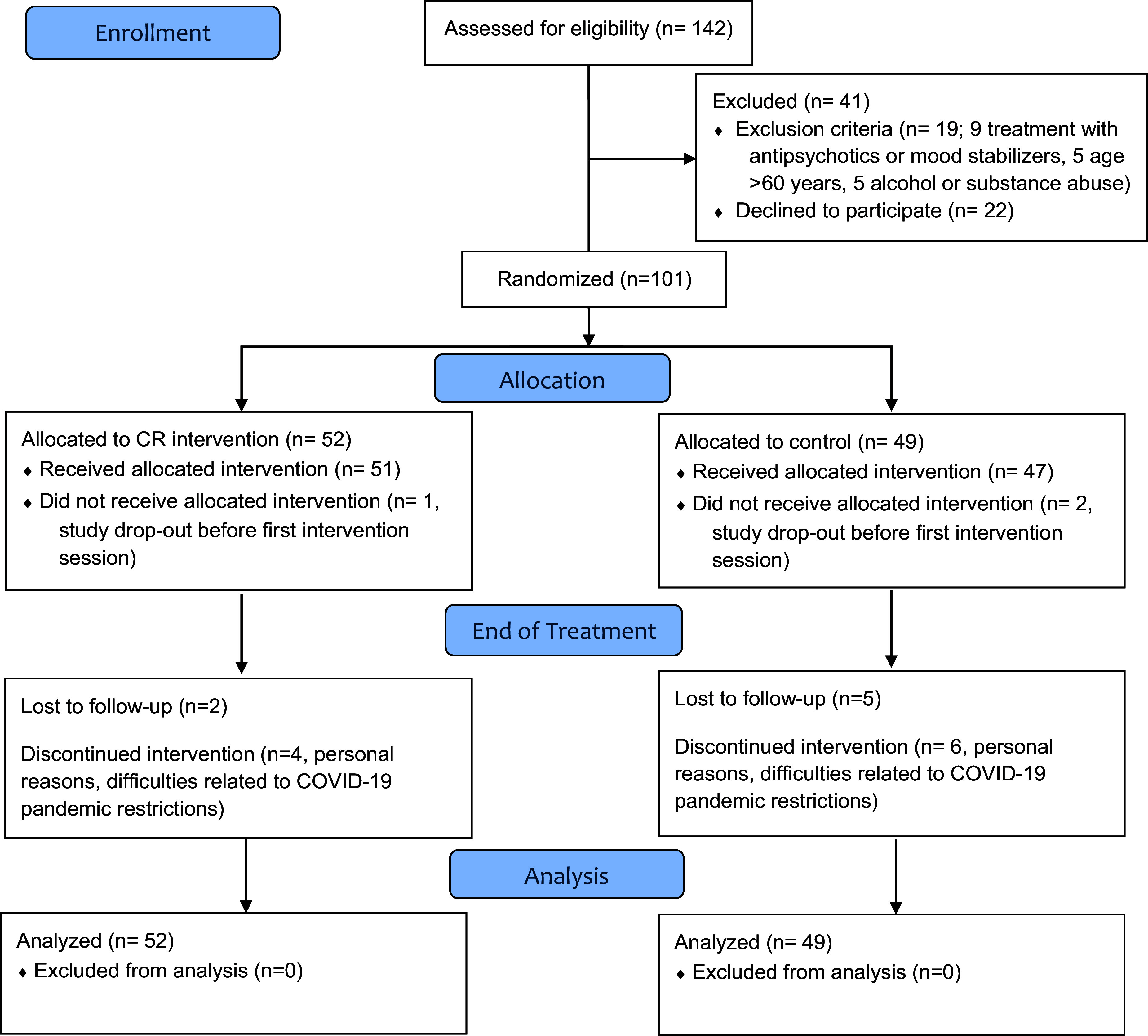
CONSORT flow diagram.

A total of 142 individuals were screened, and 101 participants were allocated to the CR group (*n* = 52) or to the CG group (*n* = 49). A total of 81 participants, 45 in the CR group and 36 in the CG group, completed the study. The complete CONSORT Flow Diagram of the study is reported in Figure 1.

Baseline socio-demographic and clinical characteristics of the total sample and between-groups comparisons are reported in Table 1. No significant between-groups differences were observed for any baseline socio-demographic or clinical characteristic.

**Table 1.** Sample characteristics and baseline comparisons

Variable	Total sample (*n* = 101) mean (± SD); *n* (%)	Cognitive remediation group (*n* = 52) mean (± SD); *n* (%)	Control condition – computer games group (*n* = 49) mean (± SD); *n* (%)	*t*-test /χ^2^-Fisher’s exact test	*p*-Value
**Age**					
(years)	38.68(±11.69)	39.40(±11.29)	37.92(±12.17)	0.636	0.526
**Gender**					
Female	34 (33.7%)	18 (34.6%)	16 (32.7%)	0.044	0.835
Male	67 (66.3%)	34 (65.4%)	33 (67.3%)		
**Education**					
(years)	13.25(±3.39)	13.13(±3.19)	13.37(±3.62)	−0.343	0.732
**Age of onset**					
(years)	29.98(±12.23)	30.88(±15.50)	29.02(±13.01)	0.764	0.447
**Severity of current MDE**					
Mild	47 (46.5%)	21 (40.4%)	26 (53.1%)	1.629	0.202
Moderate	54 (53.5%)	31 (59.6%)	23 (46.9%)		
**Duration of**	9.93(±8.71)	9.43(±8.81)	10.45(±8.66)	−0.582	0.562
**current MDE** (months)					
**Alcohol abuse** (lifetime)					
Yes	4 (4.0%)	3 (5.8%)	1 (2.0%)	–	0.618
No	97 (96.0%)	49 (94.2%)	48 (98.0%)		
**Substance abuse** (lifetime)					
Yes	7 (6.9%)	4 (7.7%)	3 (6.1%)	–	1.000
No	94 (93.1%)	48 (92.3%)	46 (93.9%)		
**Pharmacological treatment** (molecule)					
SSRI	57 (56.4%)	29 (55.8%)	28 (57.1%)	0.801	0.849
SNRI	20 (19.8%)	9 (17.3%)	11 (22.4%)		
VOR	17 (16.8%)	10 (19.2%)	7 (14.3%)		
Other^a^	7 (6.9%)	4 (7.7%)	3 (6.1%)		

Abbreviations: MDE, major depressive episode; SNRI, serotonin norepinephrine reuptake inhibitors; SSRI, selective serotonin reuptake inhibitor; VOR, vortioxetine.

a Other: amitriptyline, buproprione, clomipramine, trazodone.

Comparisons between study completers and dropouts are reported in Supplementary Table S1. No significant between-group differences in dropout rates were observed (χ^2^ = 2.713, *p* = 0.100). No differences were observed between completers and dropouts regarding baseline variables, except for psychosocial functioning as measured by the PSP (*t* = 2.296, *p* = 0.024).

Study outcomes are reported in Table 2. The CR intervention produced significantly superior results, compared to the CG control condition, in clinician-rated depressive symptoms as measured by the MADRS (*p* = 0.023, *d* = 042) and the CGI-S (*p* = 0.025, *d* = 0.39) and in subjective depressive symptoms as measured by the BDI-II (*p* = 0.005, *d* = 0.45). The CR intervention also produced significantly superior results in the working memory domain as measured by the TMT-B test (*p* = 0.004, *d* = 0.34), in the executive functions/cognitive flexibility domain with a moderate effect size, as measured by the TMT-B-A (*p* = 0.020, *d* = 0.43), and in subjective cognitive impairment as measured by the PDQ-5 (*p* = 0.006, *d* = 0.48). No significant treatment effects were observed as regards the verbal memory domain as measured by the CVLT, the processing speed domain as measured by the DSST and the TMT-A, and in real-world psychosocial functioning as measured by the PSP.

**Table 2.** Study outcomes (mixed model repeated measures)

Variable	Cognitive remediation groupT0mean (± SD)	Cognitive remediation groupT1mean (± SD)	Control condition – computer games groupT0mean (± SD)	Control condition – computer games groupT1mean (± SD)	Cognitive remediation groupTime effect*p*-Value	Control condition – computer games groupTime effect*p*-value	Time x groupF	Time x group*p*-value	Effect sizeCohen’s *d*
**MADRS**									
(score)	22.10 (±12.23)	14.04 (±10.72)	18.94 (±10.18)	15.75 (±10.39)	**<0.001**	0.140	5.326	**0.023**	0.42
**CGI-S**									
(score)	3.42 (±0.99)	2.70 (±1.03)	3.26 (±0.92)	2.92 (±0.93)	**<0.001**	0.070	5.221	**0.025**	0.39
**BDI-II**									
(score)	25.10 (±11.93)	15.18 (±11.58)	19.04 (±11.54)	14.50 (±10.86)	**<0.001**	**0.035**	8.180	**0.005**	0.45
**CVLT**									
(correct answers)	47.04 (±12.65)	52.13 (±18.33)	47.53 (±12.41)	52.89 (±14.02)	**0.029**	0.051	0.0004	0.982	0.02
**DSST**									
(correct answers)	45.29 (±13.19)	49.91 (±14.72)	48.48 (±13.79)	48.17 (±13.77)	**0.015**	0.644	1.745	0.190	0.36
**TMT-A**									
(seconds)	42.82 (±20.88)	39.54 (±21.61)	39.59 (±16.79)	35.27 (±11.36)	0.115	0.282	0.082	0.774	0.05
**TMT-B**									
(seconds)	101.20 (±67.99)	80.55 (±47.96)	83.12 (±39.05)	82.17 (±40.46)	**<0.001**	0.600	8.748	**0.004**	0.34
**TMT-B-A**									
(seconds)	58.37 (±57.15)	40.06 (±37.52)	45.57 (±31.91)	48.13 (±33.57)	**0.006**	0.509	5.687	**0.020**	0.43
**PDQ–5**									
(score)	9.13 (±4.95)	5.98 (±4.70)	6.63 (±4.49)	5.81 (±4.52)	**<0.001**	0.376	7.830	**0.006**	0.48
**PSP**									
(score)	60.67 (±18.17)	69.00 (±21.05)	63.51 (±17.26)	73.20 (±12.99)	**<0.001**	**0.006**	0.228	0.635	0.07

Abbreviations: BDI-II, Beck Depression Inventory, Second Edition; CGI-S, Clinical Global Impression - Severity scale; CVLT, California Verbal Learning Test; DSST, Digit Symbol Substitution Test; MADRS, Montgomery–Åsberg Depression Rating Scale; PDQ-5, Perceived Deficits Questionnaire – Depression 5 items; PSP, Personal and Social Performance scale; TMT, Trail Making Test. Bold font highlights significant p-values (p<0.05).

## Discussion

The present multicentric randomized controlled trial aimed to assess the effects of a CR intervention in people living with MDD undergoing a MDE.

The CR intervention showed a good feasibility profile, with a dropout rate of 13.5%: this is in line with that of other evidence-based psychosocial interventions for MDD [50, 51], as well as with that of CR interventions delivered to individuals with other diagnoses, such as Schizophrenia Spectrum Disorders [52].

The CR intervention produced significant improvements, compared to the CG control intervention, in several outcomes of interest. In particular, a larger reduction in the severity of depressive symptoms, both measured by raters and measured by the participants themselves, was observed in the CR group. The observation that CR produces significant, small-to-moderate-sized improvements in depressive symptoms is in line with the results of previous, smaller studies and of recent meta-analyses [34, 35]. The fact that this improvement is also recognized by participants, as attested by the scores of self-reported instruments, represents an encouraging finding, further confirming that CR can represent an effective intervention to treat this domain.

As regards the effects on cognitive performance, significantly small-to-moderate-sized positive effects were observed in the working memory and executive functions domains. On the contrary, no significant effect was observed in the processing speed and verbal memory domain. Again, these findings are mostly in line with those reported in the most recent meta-analytic investigation, where a positive effect was observed in the working memory and executive functions, but not in the processing speed, attention, or visual memory domains [34]. It might well be that CR interventions produce in people living with MDD undergoing a MDE improvements that are more evident in some cognitive domains, and are not capable of producing significant improvements in other domains, contrary to what can be observed in the context of CR in Schizophrenia Spectrum Disorders [29, 30]. Developing CR interventions that are specifically designed to also improve cognitive domains such as processing speed, which are among those that are more heavily interested in MDD [6, 18, 19], might represent an interesting perspective for future research.

However, considering the heterogeneity and the prevalence of cognitive impairment in depression and the substantial negative impact that it produces on the daily lives of people with MDD, where it represents one of the main predictors of functional disability [53–56], even providing small improvements in selected domains could represent a substantial benefit for participants.

As regards subjective cognitive deficits, in the present study CR produced significant, small-to-moderate-sized improvements compared to the control condition. This is an interesting and novel finding: in fact, in a recent meta-analytic investigation, a small positive effect on this outcome was observed, but the analysis did not reach statistical significance [34]; this might be due to the small number of included studies (*n* = 4) and the small sample size of each included work. Again, this finding is very encouraging, attesting that CR interventions can provide improvements that are perceived and are considered meaningful for participants: in a patient-centered and person-first approach [38], this outcome is of utmost relevance.

Conversely, no significant improvement was observed in real-world psychosocial functioning. This is a somewhat unexpected finding that could be due to different reasons. First and foremost, the lack of a follow-up observation could largely explain this result: in fact, the transfer of cognitive and clinical improvements to psychosocial functioning usually requires more time to be observed, and in samples of subjects with Schizophrenia Spectrum Disorders, longer follow-up observations are related to increased psychosocial functioning improvements [32]. Moreover, it should also be pointed out that participants who dropped out of the study showed lower baseline psychosocial functioning: this issue could have a negative impact on the results observed for this specific outcome. Finally, it should also be highlighted that part of the present study took place during the COVID-19 pandemic and this issue, which had an enormous impact on the lives of all people and in particular on those of people with mental health conditions and their daily functioning [57, 58], could have negatively impacted the transfer of cognitive gains into real world functional outcomes due to the peculiar context of the pandemic itself, with limited opportunities for novel personal interactions and developments. In any case, to better explore the impact of CR interventions on the psychosocial functioning of people with MDD, future studies should include consistent follow-up observation periods after the conclusion of the active treatment phase.

Strengths of the present study include its multicentric nature and the consequent sample size, which allows for more reliable results compared to previous individual studies, and the inclusion of participant-rated measures for both clinical and cognitive outcomes, which is becoming an increasingly important element also from a regulatory perspective [59].

A limitation of the present work is the lack of follow-up observations, which could have allowed a better evaluation of treatment impact on functional outcomes and to assess the durability of positive effects. Another limitation is the lack of direct and specific assessment of participants’ real-world outcomes: while psychosocial functioning represents an outcome of the present work and was assessed with a validated scale, instruments such as the Specific Level of Functioning [60] could allow a more nuanced and detailed evaluation of participants’ functioning.

Future perspectives include the development of CR interventions tailored specifically for the characteristics of individuals living with MDD, both during MDEs and during remission phases, the inclusion of follow-up observations even for long periods of time, the inclusion of specific measures of real-world functional outcomes, and the use of novel technologies, such as telemedicine and virtual reality to help deliver evidence-based interventions. In a clinical perspective, a wider dissemination of CR intervention in real-world, day-to-day mental health services could represent an important asset to help treat essential elements of MDD, such as cognitive impairment, providing also improvements that are perceived and appreciated directly by participants [2].

In conclusion, the results of the present work highlight that CR is an effective intervention in people with MDD, capable of improving clinical outcomes and cognitive performance both in a clinician-rated and in a subjective manner. In this perspective, CR should be considered more consistently in MDD treatment recommendations and guidelines as an evidence-based intervention and should be delivered in a more widespread manner in mental healthcare and rehabilitation services.

## Supporting information

10.1192/j.eurpsy.2025.10073.sm001 Barlati et al. supplementary material Barlati et al. supplementary material

## Data Availability

Data from the present study is available upon reasonable request to the Corresponding Author.

## Supplementary material

The supplementary material for this article can be found at http://doi.org/10.1192/j.eurpsy.2025.10073.
