# Supplementary material for: Effectiveness of cognitive remediation in subjects with major depressive disorder: A multicenter randomized controlled study in a real-world setting
Source: Eur Psychiatry. 2025 Aug 4;68(1):e106. doi: 10.1192/j.eurpsy.2025.10073 (PMC12438982; doi:10.1192/j.eurpsy.2025.10073)
Supplement: Barlati et al. supplementary material [file S0924933825100734sup001.docx]

**Supplementary Table 1.** Drop-out rates comparisons

| **Variable** | **Cognitive Remediation Group**  **n (%)** | **Control Condition – Computer Games Group**  **n (%)** | **χ²** | **p-value** |
| --- | --- | --- | --- | --- |
| Completers  Drop-outs | 45 (86.5%)  7 (13.5%) | 36 (73.5%)  13 (26.5%) | 2.713 | 0.100 |

**Supplementary Table 2.** Study completers and drop-out comparisons

| **Variable** | **Completers**  **(n=80)**  **mean (± SD)** | **Drop-out**  **(n=24)**  **mean (± SD)** | **t-test** | **p-value** |
| --- | --- | --- | --- | --- |
| **MADRS**  (score) | 19.84(±11.47) | 23.89(±10.46) | -1.374 | 0.173 |
| **CGI-S**  (score) | 21.66(±12.03) | 24.89(±12.26) | -1.024 | 0.308 |
| **BDI-II**  (score) | 3.31(±0.94) | 3.47(±1.02) | -0.656 | 0.513 |
| **CVLT**  (correct  answers) | 47.61(±12.62) | 45.84(±12.08) | 0.554 | 0.581 |
| **DSST**  (correct  answers) | 46.45(±13.56) | 48.47(±13.48) | -0.558 | 0.578 |
| **TMT-A**  (seconds) | 40.62(±19.36) | 44.00(±17.67) | -0.695 | 0.489 |
| **TMT-B**  (seconds) | 90.07(±51.97) | 102.74(±73.09) | -0.877 | 0.383 |
| **TMT-B-A**  (seconds) | 49.78(±39.81) | 62.42(±69.97) | -1.053 | 0.295 |
| **PDQ-5**  (score) | 7.84(±4.97) | 8.50(±4.55) | -0.518 | 0.606 |
| **PSP**  (score) | 63.94(±16.88) | 53.56(±19.27) | 2.296 | **0.024** |
